# Radiation-Induced Bystander Effects in Cultured Human Stem Cells

**DOI:** 10.1371/journal.pone.0014195

**Published:** 2010-12-02

**Authors:** Mykyta V. Sokolov, Ronald D. Neumann

**Affiliations:** Nuclear Medicine Division, Department of Radiology and Imaging Sciences, Clinical Center, National Institutes of Health, Bethesda, Maryland, United States of America; University of São Paulo, Brazil

## Abstract

**Background:**

The radiation-induced “bystander effect” (RIBE) was shown to occur in a number of experimental systems both in vitro and in vivo as a result of exposure to ionizing radiation (IR). RIBE manifests itself by intercellular communication from irradiated cells to non-irradiated cells which may cause DNA damage and eventual death in these bystander cells. It is known that human stem cells (hSC) are ultimately involved in numerous crucial biological processes such as embryologic development; maintenance of normal homeostasis; aging; and aging-related pathologies such as cancerogenesis and other diseases. However, very little is known about radiation-induced bystander effect in hSC. To mechanistically interrogate RIBE responses and to gain novel insights into RIBE specifically in hSC compartment, both medium transfer and cell co-culture bystander protocols were employed.

**Methodology/Principal Findings:**

Human bone-marrow mesenchymal stem cells (hMSC) and embryonic stem cells (hESC) were irradiated with doses 0.2 Gy, 2 Gy and 10 Gy of X-rays, allowed to recover either for 1 hr or 24 hr. Then conditioned medium was collected and transferred to non-irradiated hSC for time course studies. In addition, irradiated hMSC were labeled with a vital CMRA dye and co-cultured with non-irradiated bystander hMSC. The medium transfer data showed no evidence for RIBE either in hMSC and hESC by the criteria of induction of DNA damage and for apoptotic cell death compared to non-irradiated cells (p>0.05). A lack of robust RIBE was also demonstrated in hMSC co-cultured with irradiated cells (p>0.05).

**Conclusions/Significance:**

These data indicate that hSC might not be susceptible to damaging effects of RIBE signaling compared to differentiated adult human somatic cells as shown previously. This finding could have profound implications in a field of radiation biology/oncology, in evaluating radiation risk of IR exposures, and for the safety and efficacy of hSC regenerative-based therapies.

## Introduction

For many years one of the key concepts in radiation biology research posits that the direct interaction of radiation, or radiation-induced free radicals, with specific unique cellular targets (such as DNA molecules) is a necessary prerequisite for manifestation of the biological effects of ionizing radiation (IR) exposures [1]. However, about two decades ago the experimental evidence started to accumulate showing that IR could elicit secondary effects in non-irradiated cells [2]. These secondary effects, coined radiation-induced bystander effects (RIBE), are critically dependent on intercellular communication between the irradiated cells and bystanders [3], [4], [5]. These reports, and others, demonstrated that at least two independent and probably non-exclusive mechanisms of communication are involved in bystander effects, namely gap junction-mediated and secreted soluble factor-dependent signaling [6]. The ever-growing number of candidate mediators for cell culture medium-mediated bystander effects were identified, among them transforming growth factor-β (TGF- β) [7], tumor necrosis factor-α (TNF- α) [8], interleukin-6 (IL-6) [9], interleukin-8 (IL-8) [10], reactive oxygen species (ROS) [11], and reactive nitrogen species [12]. A number of bystander cell responses were reported including, but not limited to, increased yield of sister chromatid exchanges, mutations, micronucleus formation, stress response gene expression induction, terminal differentiation, apoptosis, genomic instability, transformation of cells *in vitro* and tumorigenesis *in vivo.* Bystander effects were studied in primary mammalian cell lines [5], [13], tumor cell lines [14], and, recently, in several model systems *in vivo* [15], [16]. However a comprehensive understanding of the RIBE mechanisms is still far from being complete.

Stem cell biology sparked enormous interest recently due to the mounting evidence of the key roles these cells may play in maintenance of normal tissue homeostasis, aging, and many aging-related pathologies including cancer. However, the ionizing radiation-induced bystander responses of these crucial cell populations in humans have not been systematically examined thus far. On the one hand, very little is known about RIBE in human stem cells (hSC); on the other hand, the limited data available in the literature on the bystander effect in hSC suffer from lack of consistency. For example, a recent report showed that pluripotent Oct-4-positive human embryonic stem cells (hESC) may be less susceptible to chemically-induced bystander signaling compared to adult somatic cells and spontaneously differentiated hESC [17]. Yet, human mesenchymal stem cells (hMSC) were found to exhibit the RIBE in accord with differentiated cells using intranuclear chromosomal repositioning as an endpoint [18]. In this paper we set out to mechanistically determine if human stem cells (hSC) in culture display the RIBE; and if so, what could be the underlying mechanisms of such response. We employed both medium-transfer and cell co-culture approaches to study the RIBE in two types of hSC, that is, hESC and hMSC. To assess the RIBE we looked both at the induction of DNA damage and programmed cell death (apoptosis) in bystander hSC populations. In contrast to many previous reports published thus far dealing with human adult somatic cells, we found no robust RIBE in cultured hSC. We discuss the possibilities as to why hSC might be less susceptible to RIBE signaling compared to fully differentiated cells.

## Results

### DNA damage response in bystander human mesenchymal stem cells assessed with medium transfer protocol

We and others reported that the RIBE in human adult differentiated somatic cells can be readily observed both with medium transfer and cell co-culture protocols [5], [13], [14]. For example, the magnitude of RIBE for DNA damage response (DDR) activation in WI38 human fibroblasts was 2.5-fold with medium transfer, about 3-fold with CMRA-staining cell co-culture studies, and 3.7-fold with α-particle IR exposures [5]. In order to examine RIBE in hSC grown in media collected from directly IR-exposed hSC, we performed medium transfer studies as described in the Materials and Methods section. The doses used for irradiation, the duration of conditioning of culture medium on directly IR-exposed cells, and the length of bystander incubation were based on our previous studies and data from other groups published elsewhere [5], [19], [20], [21]. Induction of DDR was determined by formation of ionizing radiation-induced foci (IRIF) in bystander cell populations, as judged by 53BP1 [22], [23] and phospho-CHK2 analysis of hSC.

**Figure 1:**
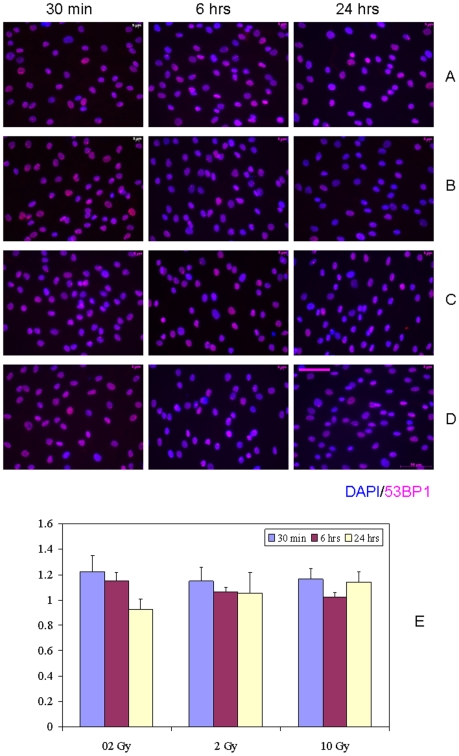
IRIF analysis of the DDR kinetics in bystander hMSC with 1 hr conditioning media transfer. (A) 0 Gy, (B) 0.2 Gy, (C) 2 Gy, and (D) 10 Gy. (E) Fraction of IRIF-positive cells in bystander hMSC population, ratios relative to sham-exposed hMSC. Legends show duration of hMSC incubation in bystander medium. Scale bar in pink equals 50 µm, 40× objective.

The fraction of nuclear IRIF-positive hMSC in bystander bulk cell populations with a 1 hr conditioned media transfer protocol showed statistically non-significant differences compared to control, sham-exposed hMSC, up to 24 hr of continuous bystander culture (Fig. 1, p>0.05). The nuclear IRIF-positive cells were defined as cells bearing at least one IRIF (either 53BP1 or phospho-CHK2). The slight increase in the proportion of bystander hMSC with activated DDR (up to 1.2 fold-change) was detected following 30 min incubation of cells with bystander media for all doses studied (Fig. 1, E). However, this effect was transient, since continuous cell incubation for 24 hr, returned the level of DDR-activated bystander hMSC to the non-irradiated control levels. No dose-dependence was observed for changes in the incidence of focus-positive hMSC over time. In our previous studies with primary human fibroblasts, we showed that the subpopulation of bystander cells bearing multiple (≥4) IRIF is significantly increased after IR exposures [5]. However, such cells were very rare in hMSC; therefore, we chose not to analyze multiple IRIF-containing hMSC as a separate subpopulation within the bulk bystander hMSC. Irradiation of media in the absence of cells and addition of these media to recipient hMSC produced no effect (data not shown). In contrast, IR induced robust DDR in hMSC and hESC, with the maximal IRIF yield seen at 30 min post-IR exposures (Fig. S1, and data not shown). The kinetics of disappearance of IRIF in hSC was typical to that observed in fully differentiated human somatic cells; i.e. the incidence of IRIF returned to near control values by 24 hr post-exposures. Also, we have seen such kinetics of DDR in hESC before in other experiments (manuscript submitted).

**Figure 2:**
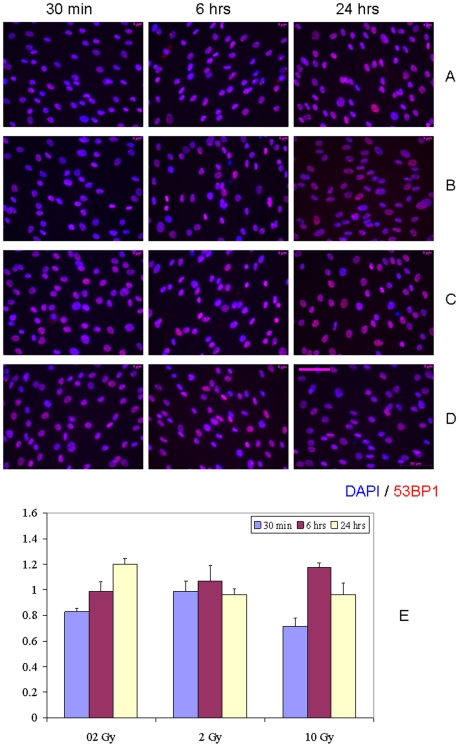
IRIF analysis of the DDR kinetics in bystander hMSC with 24 hr conditioning media transfer. (A) 0 Gy, (B) 0.2 Gy, (C) 2 Gy, and (D) 10 Gy. (E) Fraction of IRIF positive cells in bystander hMSC population, ratios relative to sham-exposed hMSC. Legends show duration of hMSC incubation in bystander medium. Scale bar in pink equals 50 µm, 40× objective.

Since the duration of bystander medium conditioning was reported to be one of the key factors affecting the bystander signaling after IR exposures [24], we analyzed the activation of DDR in bystander hMSC with 24 hr medium conditioning (Fig. 2). No statistically significant differences between sham-exposed control cultures and bystander hMSC were found for all doses we used (p>0.05). A minor drop in the yield of IRIF-positive cells occurred at 30 min of bystander culture, returning to control levels at later time points (Fig. 2, E). An exception occurred at the 0.2 Gy dose, for which some increase in DDR (1.2– fold) in bystander hMSC was seen up to 24 hr; however, the effect observed was not statistically significant (p>0.05).

### DNA damage response in bystander human embryonic stem cells assessed with medium transfer protocol

**Figure 3:**
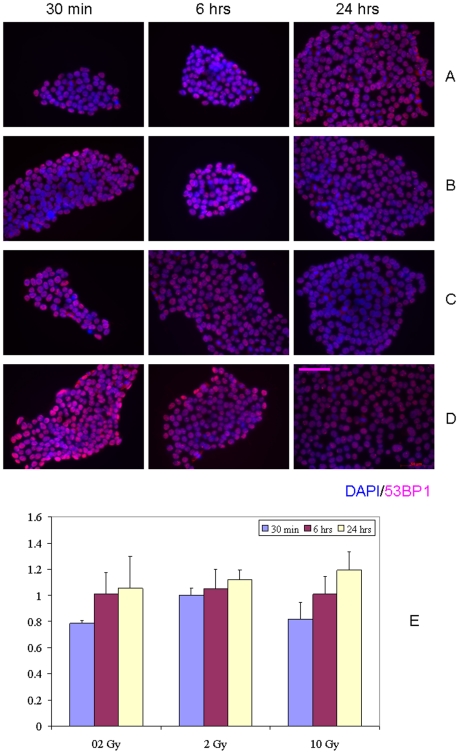
IRIF analysis of the DDR kinetics in bystander hESC with 1 hr conditioning media transfer. (A) 0 Gy, (B) 0.2 Gy, (C) 2 Gy, and (D) 10 Gy. (E) Fraction of IRIF positive cells in bystander hESC population, ratios relative to sham-exposed hESC. Legends show duration of hESC incubation in bystander medium. Scale bar in pink equals 50 µm, 40× objective.

**Figure 4:**
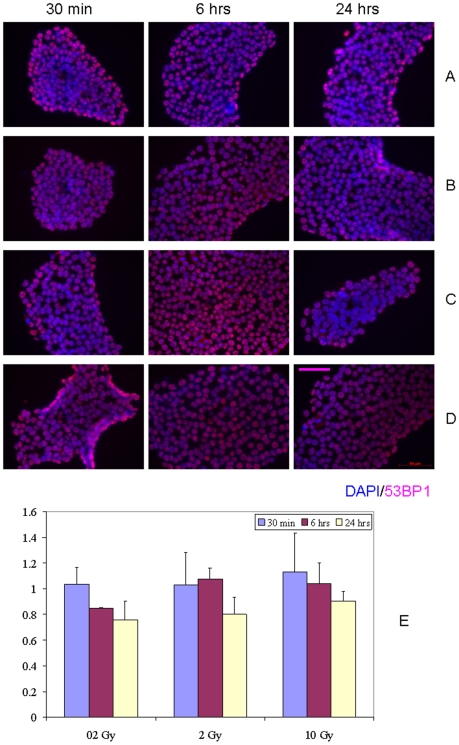
IRIF analysis of the DDR kinetics in bystander hESC with 24 hr conditioning media transfer. (A) 0 Gy, (B) 0.2 Gy, (C) 2 Gy, and (D) 10 Gy. (E) Fraction of IRIF positive cells in bystander hESC population, ratios relative to sham-exposed hESC. Legends show duration of hESC incubation in bystander medium. Scale bar in pink equals 50 µm, 40× objective.

In the next step of our studies, we sought to evaluate whether DDR is activated as a result of RIBE in hESC. Towards this end, we irradiated hESC (H9 cell line) with either 0.2 Gy, 2 Gy or 10 Gy of X-rays, allowed the cells to incubate for either 1 hr or 24 hr, and then harvested the conditioned medium and transferred it to recipient bystander hESC. We observed no increases in DDR induction in bystander hESC both after 1 hr or 24 hr of medium conditioning for all doses and incubation times studied (Fig. 3 and Fig. 4, p>0.05). Curiously, for some combinations of doses and incubation times there was a drop in the fraction of IRIF-positive cells in bystanders compared to control cell cultures (0.2 Gy and 30 min of bystander culture, Fig. 3; 0.2 Gy and 6 hr, 0.2 Gy and 24 hr of bystander culture, Fig. 4), although the observed effect was subtle (0.8– fold change). Irradiation of media in the absence of hESC demonstrated no effect in recipient hESC cultures (data not shown).

### Induction of DNA damage response in bystander human mesenchymal stem cells co-cultured with directly irradiated autologous cells

**Figure 5:**
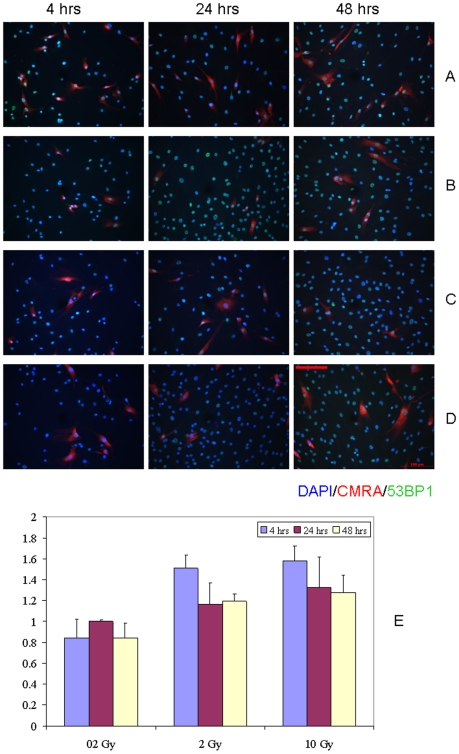
IRIF analysis of the DDR kinetics in bystander hMSC co-cultured with directly IR-exposed hMSC. (A) 0 Gy, (B) 0.2 Gy, (C) 2 Gy, and (D) 10 Gy. (E) Fraction of IRIF positive cells in bystander hMSC population, ratios relative to sham-exposed hMSC. Shown in legends is the duration of hMSC co-culture incubation. Scale bar in red equals 100 µm, 20× objective.

The results of our previous studies indicate that the magnitude of the bystander effect is maximal under the conditions of continuous co-cultivation of directly IR-exposed and bystander cells [5]. Therefore, we evaluated the DDR activation in hMSC co-cultured with CMRA-labeled IR-exposed hMSC (as described in the Materials and methods section). hMSC labeling with CMRA resulted in no statistically significant changes in IRIF as compared to non-stained hMSC (data not shown). We found no statistically significant changes in the proportion of IRIF-positive bystander hMSC compared to sham-irradiated cell co-culture control populations (Fig. 5, p>0.05). However, a modest transient increase in the yield of bystander cells with activated DDR was evident at 4 hr of co-culture following both 2 Gy and 10 Gy IR exposures (about 1.5– fold over control). This effect was not persistent; starting from 24 hr of co-culture the level of IRIF-positive cells returned to control values in bystander hMSC cultures (Fig. 5, E).

### Induction of apoptotic cell death in bystander human stem cells

**Figure 6:**
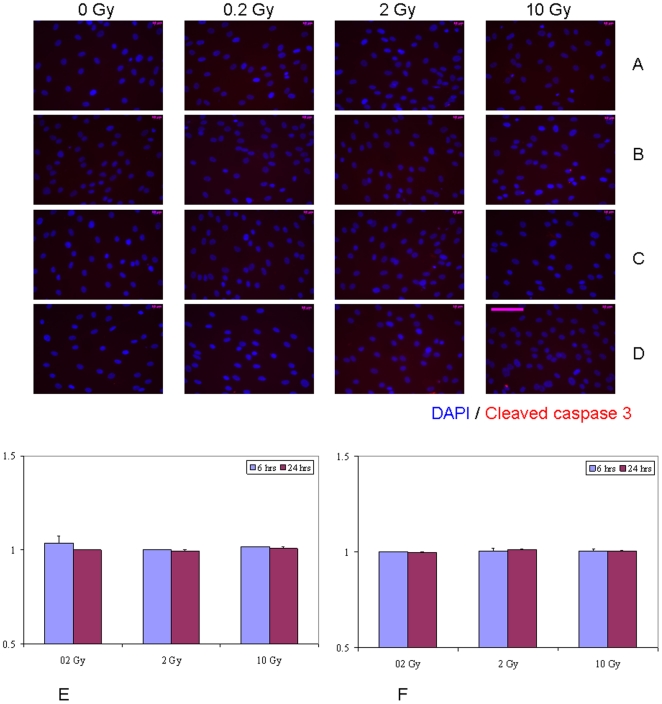
Immunofluorescence analysis of the kinetics of apoptotic death in bystander hMSC with media transfer protocol. (A, B) Bystander medium harvested from directly irradiated hMSC 1 hr post-exposure; (C, D) bystander medium harvested from directly irradiated hMSC 24 hr post-exposure. Bystander hMSC were cultured in conditioned medium either for 6 hr (A, C) or for 24 hr (B, D). Stained in red - cleaved caspase 3– positive apoptotic hMSC; in blue – hMSC nuclei (DAPI). Fraction of cleaved caspase 3 positive hMSC in cell populations grown in bystander medium conditioned on IR-exposed cells for either 6 hrs (E) or 24 hrs (F), ratios relative to sham-exposed hMSC. Legends show duration of incubation in bystander media. Scale bar in pink equals 50 µm, 40× objective.

**Figure 7:**
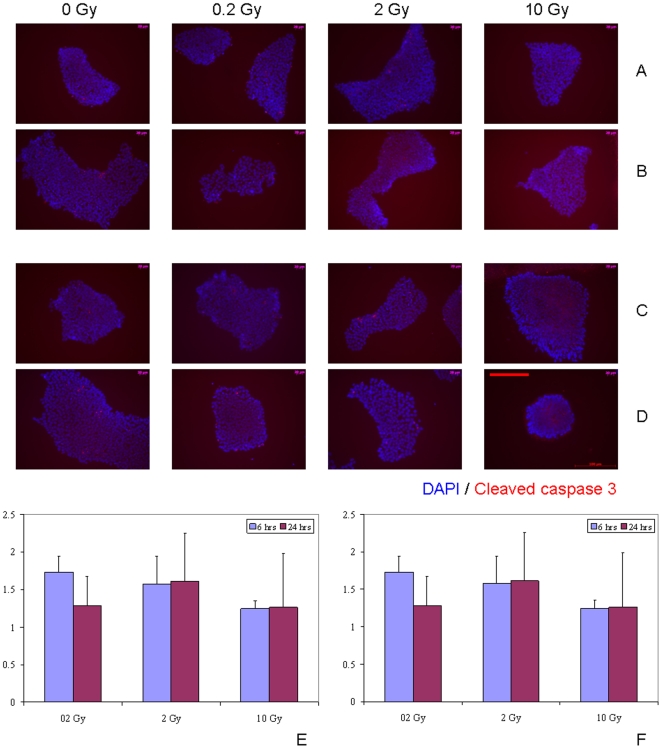
Immunofluorescence analysis of the kinetics of apoptotic death in bystander hESC with media transfer protocol. (A, B) Bystander medium harvested from directly irradiated hESC 1 hr post-exposure; (C, D) bystander medium harvested from directly irradiated hESC 24 hr post-exposure. Bystander hESC were cultured in conditioned medium either for 6 hr (A, C) or for 24 hr (B, D). Stained in red - cleaved caspase 3 – positive apoptotic hESC; in blue – hESC nuclei (DAPI). Fraction of cleaved caspase 3 positive hESC in cell populations grown in bystander medium conditioned on IR-exposed cells for either 6 hrs (E) or 24 hrs (F), ratios relative to sham-exposed hESC. Legends show duration of incubation in bystander media. Scale bar in red equals 100 µm, 20× objective.

It is widely accepted that one of the key hallmarks of the RIBE signaling is the increase in the apoptotic cell death in exposed cell populations both *in vitro* and *in vivo* [25], [26], [27]. Hence, we set out to determine whether the bystander medium harvested from directly irradiated hSC elicits such a response. The apoptotic cell death was negligible in directly IR-exposed hMSC (Fig. S2). No evidence of induction of apoptotic cell death was seen in hMSC grown for either 6 hr or 24 hr in bystander medium conditioned for either 1 hr or 24 hr (Fig. 6 and Fig. S3). In contrast, massive programmed cell death occurred in hESC after both 2 Gy and 10 Gy IR exposures (Fig. S4) that was evident at 6 hr post-IR. Although these cells tend to easily undergo apoptosis as a result of even slightly suboptimal cell culture conditions, we observed a lack of robust apoptosis-inducing RIBE in hESC (Fig. 7, E-F, p>0.05 and Fig. S5). Only 6 hr of cultivation of bystander hESC in medium conditioned for 1 hr following irradiation of hESC with 0.2 Gy revealed modest (1.5– fold change) increase in the apoptotic cell death compared to sham-exposed cells (Fig. 7, E). However, given that only about 1.5–2% of hESC in control, non-irradiated cell populations underwent apoptosis at any given time in our studies, the biological significance of such modest RIBE affecting 2–3% of cells is questionable. Moreover, this effect was not persistent since at 24 hr of incubation no increase in apoptosis was observed for any of experimental conditions examined.

### DNA damage response in bystander human mesenchymal stem cells grown in medium conditioned on irradiated human somatic non-stem cells

**Figure 8:**
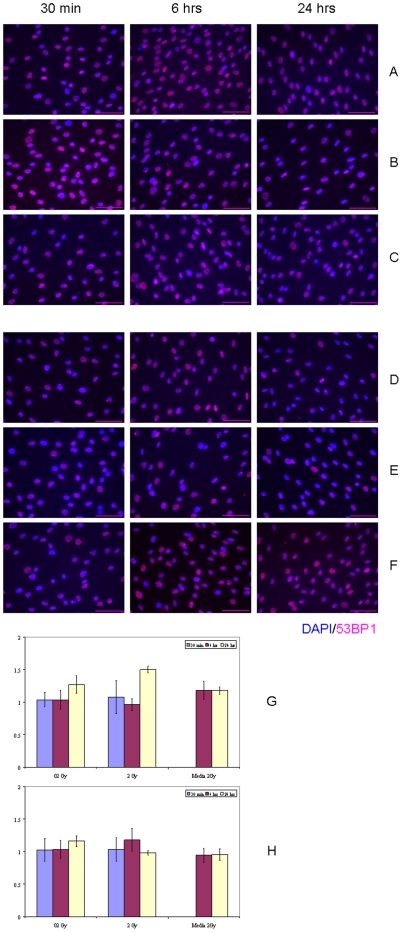
IRIF analysis of the DDR kinetics in bystander hMSC with 1 hr conditioning media transfer using IR-exposed human non-stem cell cultures. (A–C) IMR-90 cell cultures were exposed to (A) 0 Gy, (B) 0.2 Gy, and (C) 2 Gy. (D–F) T98G cell cultures were exposed to (D) 0 Gy, (E) 0.2 Gy, and (F) 2 Gy. The medium was conditioned for 1 hr post-IR exposures, then harvested, filtered as described in Materials and Methods, and transferred to bystander hMSC for the indicated time points. (G) Fraction of IRIF-positive cells in bystander hMSC population grown in IMR-90 conditioned medium, ratios relative to sham-exposed hMSC. (H) Fraction of IRIF-positive cells in bystander hMSC population grown in T98G conditioned medium, ratios relative to sham-exposed hMSC. Legends (G–H) show duration of hMSC incubation in bystander medium. Scale bar in pink equals 50 µm, 40× objective.

Since it has been shown that human bone-marrow derived MSC could migrate to the sites of injury [28], we set out to test if irradiated human non-stem cells could elicit a RIBE in hMSC. We examined the DDR activation in hMSC cultivated with media conditioned on either IMR-90 normal human lung fibroblasts or glioblastoma T98G cells exposed to 0.2 Gy or 2 Gy of X-rays (Fig. 8). No significant increase in DDR induction was observed for experimental conditions studied, except for one timepoint (Fig. 8, G, p>0.05). Even after 24 hr of hMSC incubation in bystander medium collected 1 hr after 2 Gy IR exposures of IMR-90 cell cultures, the magnitude of RIBE was only about 1.5-fold increase over sham-treated hMSC. In marked contrast, under the same experimental condition, transfer of bystander medium from 2 Gy IR-exposed IMR-90 cultures to bystander IMR-90 cultures for 24 hrs resulted in a profound RIBE with a magnitude about 4.5-fold increase over sham-treated IMR-90 cells (Fig. S6). This finding is in concert with our previous work in which we observed RIBE in human WI-38 fibroblasts for the same endpoint [5]. With this positive control for RIBE in human non-stem cells, we ascertained that our IRIF-based assay was sensitive enough to detect RIBE in human cells, and that human SC might be less susceptible to RIBE than human somatic differentiated cells.

## Discussion

Human stem cells are thought to be the root of the hierarchical organization of many, if not all, organs and tissues within the organism; and, therefore, play a key role in maintenance of tissue homeostasis thus critically contributing to human overall well-being. At the same time hSC are considered to be particularly vulnerable to deleterious effects of endogenous and exogenous stress exposures [29]. In fact, many adult hSC reside in their niches for prolonged time and might be susceptible to an increased DNA mutation load as a result of accumulation of DNA damage over time. Some reports imply that so-called tumor stem cell (TSC) or tumor-initiating cells may arise from transformed normal adult stem cells [30]. A growing body of evidence suggests that DNA damage can be caused not only by exposure to genotoxins, including IR, *per se*, but also as a result of non-targeted effects of IR like RIBE [5], [13], [14]. An ever increasing number of studies points to the fact that RIBE is a complex, multifaceted phenomena that is observed both *in vitro* and *in vivo*; and, as such, might be of importance not only to the broad field of radiation biology, but also of relevance in implementation of novel therapeutic regimens in radiation oncology to treat cancer patients [31]. However, surprisingly little is known how crucial hSC compartment respond to RIBE. To address this issue, we undertook the current study. The goal of the work presented here was to mechanistically interrogate RIBE in hSC, and to determine the possible mechanisms underpinning this phenomenon. The non-targeted effects of IR (including RIBE) are generally considered to be low-dose phenomena; however, recent reports suggest that doses of IR exposures up to 10 Gy can elicit RIBE in human cells [20], [21]. Therefore, we set out to comprehensively evaluate RIBE in hSC using relatively low (0.2 Gy), clinically relevant single fraction (2 Gy) and high dose (10 Gy) IR exposures. Timings of medium conditioning and bystander incubation were chosen based on our previous data and the results of other groups published elsewhere [5], [14], [20].

We and others reported that the RIBE in human adult differentiated somatic cells can be readily observed both with medium transfer and cell co-culture protocols [5], [13], [14]. For example, the magnitude of RIBE for DDR activation in WI38 human fibroblasts was 2.5-fold with medium transfer, about 3-fold with CMRA-staining cell co-culture studies, and 3.7-fold with α-particle IR exposures [5]. With the other types of human cells, we observed the magnitude of RIBE to be no less than 2-fold over sham-exposed cells with bystander medium transfer technique [14]. Moreover, in the artificial human 3-D tissue models, the magnitude of α-particle induced-RIBE was a 4- to 6-fold increase over control values [32]. In previous studies, the RIBE usually peaked at 1–2 days after IR exposures [5], then gradually decreased over a 7-day time course [32]. However, cultured hSC in our present work fail to exhibit such robust RIBE for both DDR activation and cell death endpoints to an extent observed with human differentiated cells. Only the subtle changes such as 1.2-fold increase in DDR for hMSC with bystander media transfer, and 1.5-fold increase in DDR in hMSC co-cultured with directly IR-exposed hMSC at the earliest timepoints studied (30 min and 4 hr, respectively) suggest there is very little RIBE in hSC. RIBE is thought to be detrimental to cells; hence, one of the possible explanations for our findings is that hSC possess enhanced ability to cope with stressful conditions [33]. In fact, several lines of research imply that the antioxidant system and DNA repair ability of normal hSC and TSC are superior to that found in more differentiated progeny of hSC [34], [35], [36]. It may be argued that hSC are not vulnerable to non-targeted effects of IR exposures based on the concept of RIBE as being a part of a more generalized cellular stress response; a view which is gaining momentum recently [14], [37]. Other possibility is that hSC are merely not competent to receive RIBE signals, or, alternatively, hSC are not producing RIBE signals. We tested this possibility by examining the RIBE in hMSC receiving the bystander medium collected from IR-exposed human somatic non-stem cells, that is, IMR-90 and T98G cell lines reported in the literature being producers of strong bystander signals [10], [38], [39], [40], [41]. Whereas bystander IMR-90 showed a robust RIBE in medium transfer experiments, hMSC exposed to bystander IMR-90 medium failed to do so. The absence of RIBE, at least in some well-defined experimental system models, is becoming increasingly appreciated by scientific community [42], [43], [44], [45]. One of the most recent observations suggest that bystander signals could potentially be sensitive to light exposure during cell culture handling, and serum batch may play a significant role in manifestation of RIBE [46]. However, this factor is unlikely involved in our present study, since we performed cell culture media transfer manipulations in dim light. Moreover, the same batch of serum gave a strong RIBE signal in experiments with IMR-90 cell cultures, but failed to do so in hMSC populations. Why in some cases RIBE is so robust, while in others including our current study in hSC, it is virtually absent, is still not clear and definitely merits further investigations. It is highly unlikely that the lack of detrimental outcomes of RIBE we found in our hSC experiments was due to non-adequate endpoints chosen for our analyses, since cell killing and DDR activation are among the most extensively used parameters to assess RIBE by others. A single published study demonstrating RIBE in hMSC was based upon a different endpoint for evaluation of this phenomenon; i.e., the intranuclear movement of pericentromeric loci of 1q12 chromosomes from the nuclear membrane towards the nuclear center [18]. The biological significance of this translocation, and how it affects the ultimate fate of bystander hMSC, is unclear from that work. Interestingly, the involvement of some unanticipated molecules practically absent from the vast majority of human somatic cells, such as telomerase, was shown recently to be important in mediating the susceptibility to non-targeted intercellular communication effects under stressful conditions. Pluripotent hESC expressing high levels of telomerase were non-responsive to chemically induced bystander signaling, but hESC undergoing differentiation showing increased vulnerability to such signaling [17]. In our studies, pluripotent hESC positive for telomerase showed no evidence for RIBE; however, the same was true for multipotent hMSC which are expressing telomerase at very low levels (data not shown). Accordingly, there is currently no strong evidence to suggest any particular importance of telomerase mediating resistance to RIBE in hSC. But, it will be of interest to elucidate whether the hSC undergoing directed differentiation along a specific lineage remain tolerant to RIBE; and, if not, what the underlying mechanisms responsible for such a possible shift may be.

Human stem cells in vivo reside in a specific microenvironments or niches which interact with hSC to ultimately regulate cell fate. Stem cell niches are generally thought to maintain hSC in a quiescent state somewhat outside regular tissue control to maintain their genomic stability, pluripotency and/or help to evade proliferation/differentiation signals that may emanate from the bulk tissue [47]. Cell-cell interactions between hSC within the niche are one of the key factors involved in the body homeostasis [48], therefore, in the present study we set out to address the issue of how irradiated hSC could potentially affect bystander hSC. The other factor maintaining stem cell identity and considered to be important for hSC regulation is interaction of hSC and non-stem cells. This aspect of hSC biology is especially interesting since hMSC are known to actively migrate to compartments comprised of non-stem cells and interact with them upon injury [28] and/or diseased states, such as cancer [49]. We interrogated these interactions using bystander media transfer technique with hMSC and irradiated non-stem normal and cancerous cells. The interactions between hSC and extracellular matrix components, growth factors, hormones, physiochemical properties of microenvironment are adding to a complexity of mutual interplay within hSC and their niches within the human body. An issue that remains to be explored is the translation of our findings using cultured hSC *in vitro* to the situation *in vivo*. If hSC within their niches in the body turn out to be not susceptible to RIBE, it can provide some additional flexibility in administration of radiation therapies that may potentially affect normal tissues producing unwanted side-effects of a treatment. Hence, the RIBE in general [50], and the finding of a lack of RIBE in hSC, in particular, might be of a direct relevance to cancer therapy. An additional layer of complexity is added by the studies arguing that RIBE is potentially genotype-dependent [51], [52], [53]. The selection of experimental tissue specimens from people with different genomic backgrounds might help to clarify this point in further studies. The RIBE is known to be triggered by both high-LET and low-LET IR exposures; however, there are reports showing that in some cases RIBE can be observed only under very specific conditions of IR exposures, and as such, appears to be highly variable [44]. Even taking into account these limitations and uncertainties associated with RIBE in hSC, the findings emerging from our present work provide a conceptual framework to study RIBE in hSC. However, it is presently unclear if the data on RIBE in hSC would significantly affect evaluation of radiation risks stemming from human IR exposures since the risk estimates are mostly based on epidemiology data which presumably includes the contribution of the RIBE to risk. Future studies will need to address these issues in more detail.

## Materials and Methods

### Human mesenchymal stem cells

Human bone-marrow derived human mesenchymal stem cells (Lonza, Poietics Stem Cells, PT-2501) were used between passages 4–5. According to manufacturer's, these cells were positive for CD105, CD166, CD29, and CD44; cells tested negative for CD14, CD34 and CD45 at the initiation of cell culture. The cells were grown in Mesenchymal Stem Cell Growth Medium (MSCGM, Lonza, PT-3001, with added L-glutamine and mesenchymal cell growth supplement) that was specifically formulated for growing large numbers of mesenchymal stem cells without inducing differentiation. Cell cultures were fed with fresh growth media every 3–4 days, grown to 70%–80% confluence, and then subcultured with Trypsin-EDTA (Lonza), per supplier's protocol. A total of 5×10^3^ cells were routinely plated per each cm^2^ of cell culture vessel surface upon passaging.

### Human embryonic stem cells

Human embryonic stem cells (H9, WiCell) were used between passages 32–36. They were routinely cultured in mTeSR-1 medium (Stemcell Technologies) on a BD Matrigel hESC-qualified matrix (BD Biosciences) at 37°C and 5% CO_2_. Cell cultures were maintained and propagated following supplier's protocol. Cells were passaged every 5–7 days using collagenase IV (Invitrogen). The medium was changed every day, per suppliers' protocol.

### Human somatic non-stem cells

Human IMR-90 normal lung fibroblast cells (Coriell Cell Repositories, Camden, NJ) were used between passages 8–11. The cell cultures were maintained in Eagle Minimum Essential Medium with Earle's Balanced Salt Solution (EMEM, ATCC, Manassas, VA) and subcultured with Trypsin-EDTA (Invitrogen, Carlsbad, CA). Human glioblastoma T98G cells were obtained from ATCC and cultured in RPMI1640 (Invitrogen, Carlsbad, CA).

### Bystander treatment - medium transfer

Cell cultures grown to about 70% confluence were either exposed to X-ray radiation with X-RAD 320 Biological Irradiator unit (Precision X-Ray, Inc.; dose rate about 1 Gy/min; 320 kV, 12.5 mA), or were sham-irradiated. Doses of irradiation used were 0.2 Gy, 2 Gy or 10 Gy. Then cell cultures were allowed to recover in CO_2_ incubator for either 1 h or 24 h. The conditioned medium (CM) samples were harvested, passed through 0.22 µm MILLEX GP filters (Millipore) and transferred to bystander cell cultures for 30 min, 6 h or 24 h for analysis of RIBE using various endpoints. The manipulations were performed in dim light. Media transferred from sham-exposed cultures and media irradiated in the absence of cells were used as controls.

### Bystander treatment – cell co-culture protocol

Human MSC cultures were seeded in Labtek II four-well glass slides (Nalge Nunc International) at 1×10^4^ cells per well. After overnight growth, 5 µM CMRA dye (Invitrogen) was added to selected subsets of cell cultures grown on multiwell slides for 30 min, per the manufacturer's protocol. The cells were then incubated for 30 min in freshly added regular media. On a same day, the cultures on multiwell slides were exposed to 0.2 Gy, 2 Gy or 10 Gy of X-ray radiation (X-RAD 320 Biological Irradiator unit, Precision X-Ray, Inc.; dose rate about 1 Gy/min; 320 kV, 12.5 mA), or were sham-irradiated at ambient temperature. In parallel, hMSC were grown in T175 flasks, so that cell cultures reached about 70% confluence on a day of experiment. The T175 cultures were trypsinized and 1×10^3^ cells were added to the irradiated cultures immediately after IR exposures. The mixed cell cultures (co-cultures) were incubated for 4 h, 24 h or 48 h before downstream analysis.

### Immunocytochemistry— IR-induced focus formation assay

The IR-induced focus (IRIF) formation assay was carried out using a protocol described previously [5], [14]. The cell cultures were fixed and blocked with 3% bovine serum albumin (Sigma). Fixed cells were incubated for 1 h at room temperature with primary antibodies, rabbit polyclonal to human 53BP1 (1∶250) (Santa Cruz Biotechnology) or with rabbit polyclonal to human phospho-Thr68-CHK2 (Calbiochem). The cells were overlaid with secondary Alexa555-conjugated antibodies (1∶500) (Invitrogen). After several washes, nuclei were counterstained with DAPI, coverslips were mounted using mounting medium with antifade (VectaShield, Vector Labs). The samples were examined by Axioplan2 Zeiss fluorescence microscope (Carl Zeiss) with the camera image acquisition parameters set constant throughout experiments. Filter settings were set as follows: DAPI channel - 18, TRITC channel –500 (40× objective). In cases images were taken using 20× objective, the filter settings were as below: DAPI channel - 100, TRITC channel –2200. A few hundred cells were scored for each datapoint; the bystander cells containing at least one readily discernable by eye IRIF were enumerated, and were considered to be IRIF-positive.

### Annexin V assay

The bystander cell populations were assessed for the incidence of apoptotic cells. To this end, Annexin V assay identifying the translocation of phosphatidylserines from the inner surface to the outer leaflet of the cell plasma membrane during the early stages of apoptosis was used (Annexin V-EGFP Apoptosis Detection kit, Genscript), per manufacturer protocol. Briefly, the bystander cell cultures were washed twice with PBS. Then, 5 µl Annexin V-EGFP and 5 µl propidium iodide (PI) were added to 500 µl Binding Buffer, mixed and incubated with cell cultures for 5 min, in the dark.

The cultures were observed under Axiovert 200M Zeiss fluorescence microscope (Carl Zeiss) using a dual filter for FITC and rhodamine. Cells bound by annexin V-EGFP appeared to have green plasma membranes (early apoptotic cells). Cells that lost membrane integrity were observed to have nuclei stained in red (PI) and a halo of green on the cell plasma membrane (late apoptotic cells).

### The caspase-3 assay

The cleavage of one of the major executioner caspases, namely, caspase-3 in apoptotic cells is known to represent a point-of-no-return in a chain of intracellular events leading to programmed cell death. The evaluation of this relatively late event in apoptotic signaling pathway was done using immunocytochemical staining in bystander stem cells with cleaved caspase-3 specific monoclonal antibody (Cell Signaling, Inc.). The protocol was followed as described in [54].

### Statistical analysis

Data from at least three independent experiments/measurements were calculated and presented in paper's Figures as means and standard errors of the mean. The Students' t-tests were used to compare the results from irradiated and mock-treated cell cultures. The differences between groups were considered significant if the p-value was less or equal to 0.05.

## Supporting Information

Figure S1 IRIF analysis of the DDR kinetics in hMSC irradiated with (A) 0 Gy, (B) 0.2 Gy, (C) 2 Gy, and (D) 10 Gy. DAPI-stained cell nuclei are in blue, and 53BP1 staining is in red. Scale bar in pink equals 50 µm, 40× objective.
(0.89 MB TIF)

Figure S2 Live cell analysis of the kinetics of apoptotic cell death in directly IR-exposed hMSC. Irradiated hMSC were cultured either for 6 hr (A) or for 24 hr (B) post-IR. Shown in green are Annexin V-positive (apoptotic) hMSC, in red - apoptotic hMSC nuclei stained with PI. Scale bar in white equals 100 µm, 10× objective.
(0.55 MB TIF)

Figure S3 Analysis of the kinetics of apoptotic death in bystander hMSC with media transfer protocol. (A, B) Bystander medium harvested from directly irradiated hMSC 1 hr post-exposure; (C, D) bystander medium harvested from directly irradiated hMSC 24 hr post-exposure. Bystander hMSC were cultured in conditioned medium either for 6 hr (A, C) or for 24 hr (B, D). Stained in green are Annexin V-positive apoptotic hMSC; in red - PI-stained nuclei of apoptotic hMSC. Scale bar in white equals 100 µm, 10× objective.
(0.94 MB TIF)

Figure S4 Live cell analysis of the kinetics of apoptotic cell death in directly IR-exposed hESC. Irradiated hESC were cultured either for 6 hr (A) or for 24 hr (B) post-IR. Shown in green are Annexin V-positive (apoptotic) hESC, in red - apoptotic hESC nuclei stained with PI. Scale bar in white equals 100 µm, 10× objective.
(0.55 MB TIF)

Figure S5 Analysis of the kinetics of apoptotic cell death in bystander hESC with media transfer. (A, B) Bystander medium harvested from directly irradiated hESC 1 hr post-exposure; (C) bystander medium harvested from directly irradiated hESC 24 hr post-exposure. Bystander hESC were cultured in conditioned medium either for 6 hr (A, C) or for 24 hr (B). Shown in green are Annexin V-positive (apoptotic) hESC, in red - apoptotic hESC nuclei stained with PI. Scale bar in white equals 100 µm, 10× objective.
(0.82 MB TIF)

Figure S6 IRIF analysis of the DDR activation in bystander IMR-90 cells receiving medium conditioned for 1 hr on IMR-90 irradiated with either (A) 0 Gy, or (B) 2 Gy. Shown is the magnitude of RIBE (fraction of cells with no less than 4 IRIF per nucleus) relative to sham-irradiation, expressed as mean value ± SEM. DAPI-stained cell nuclei are in blue, and 53BP1 staining is in red. Scale bar in pink equals 50 µm, 40× objective.
(0.60 MB TIF)
