# Supplementary figures and images for: Radiation-Induced Bystander Effects in Cultured Human Stem Cells
Source: PLoS One. 2010 Dec 2;5(12):e14195. doi: 10.1371/journal.pone.0014195 (PMC2996280; doi:10.1371/journal.pone.0014195)

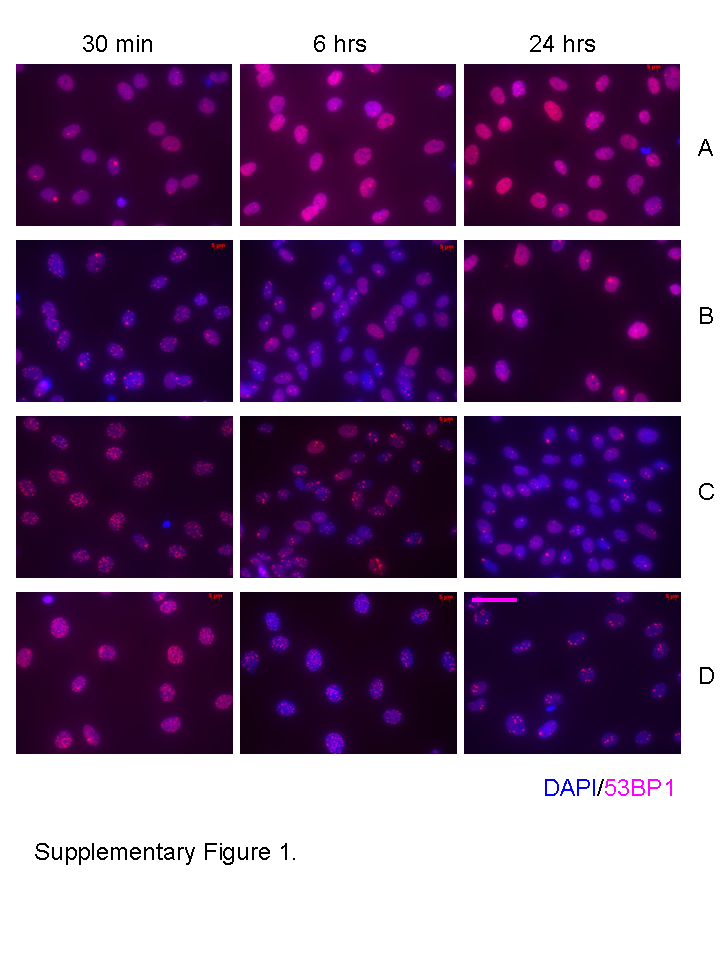

Supplement: Figure S1 — IRIF analysis of the DDR kinetics in hMSC irradiated with (A) 0 Gy, (B) 0.2 Gy, (C) 2 Gy, and (D) 10 Gy. DAPI-stained cell nuclei are in blue, and 53BP1 staining is in red. Scale bar in pink equals 50 µm, 40× objective. (0.89 MB TIF) [file pone.0014195.s001.tif]

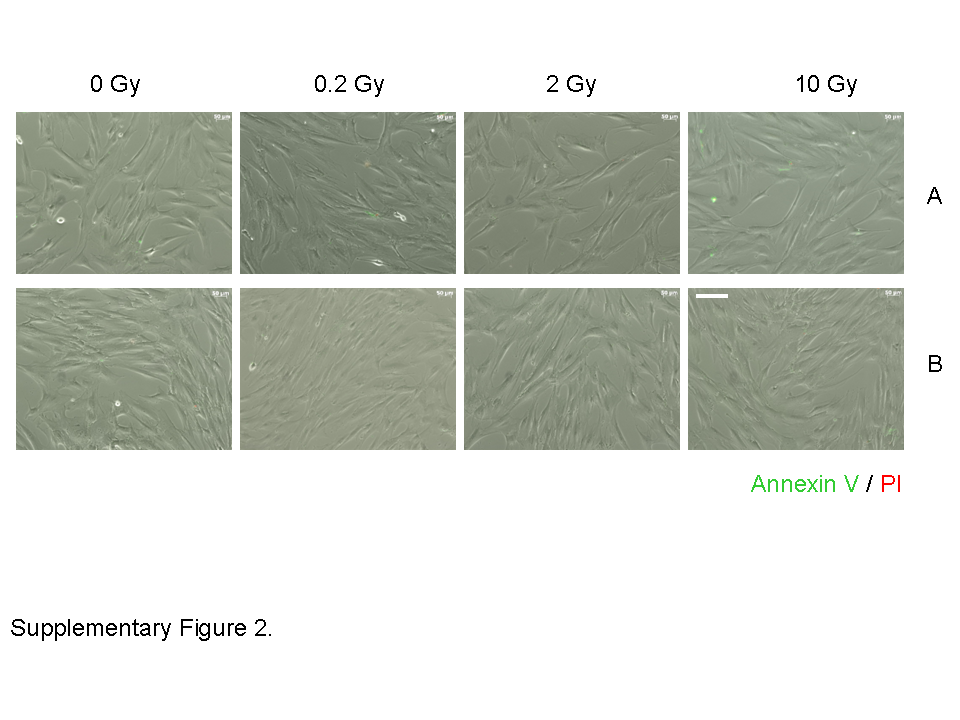

Supplement: Figure S2 — Live cell analysis of the kinetics of apoptotic cell death in directly IR-exposed hMSC. Irradiated hMSC were cultured either for 6 hr (A) or for 24 hr (B) post-IR. Shown in green are Annexin V-positive (apoptotic) hMSC, in red - apoptotic hMSC nuclei stained with PI. Scale bar in white equals 100 µm, 10× objective. (0.55 MB TIF) [file pone.0014195.s002.tif]

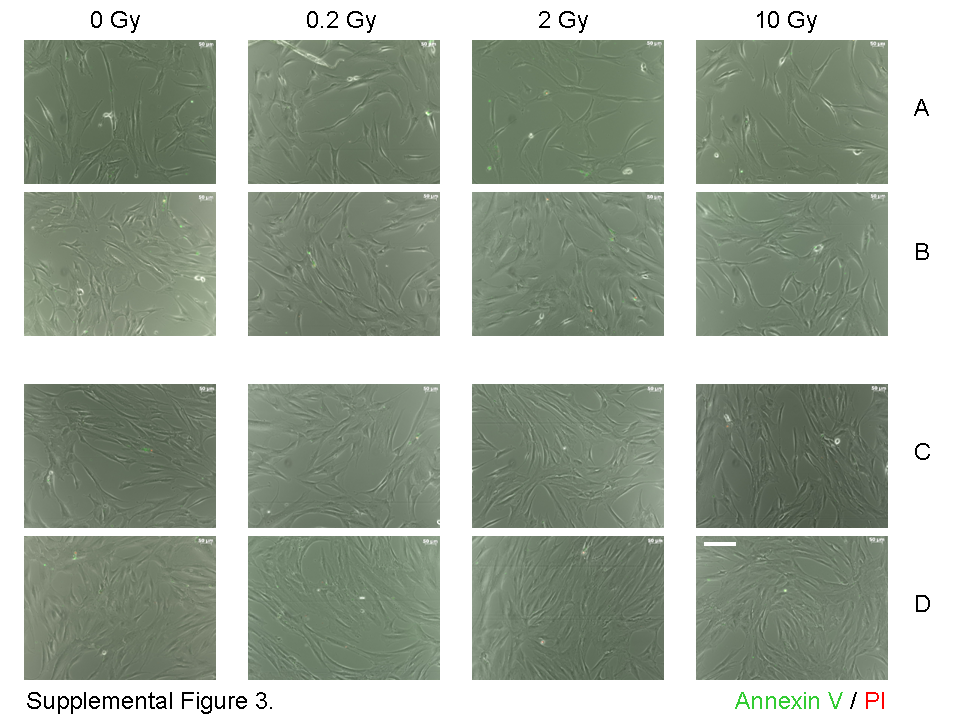

Supplement: Figure S3 — Analysis of the kinetics of apoptotic death in bystander hMSC with media transfer protocol. (A, B) Bystander medium harvested from directly irradiated hMSC 1 hr post-exposure; (C, D) bystander medium harvested from directly irradiated hMSC 24 hr post-exposure. Bystander hMSC were cultured in conditioned medium either for 6 hr (A, C) or for 24 hr (B, D). Stained in green are Annexin V-positive apoptotic hMSC; in red - PI-stained nuclei of apoptotic hMSC. Scale bar in white equals 100 µm, 10× objective. (0.94 MB TIF) [file pone.0014195.s003.tif]

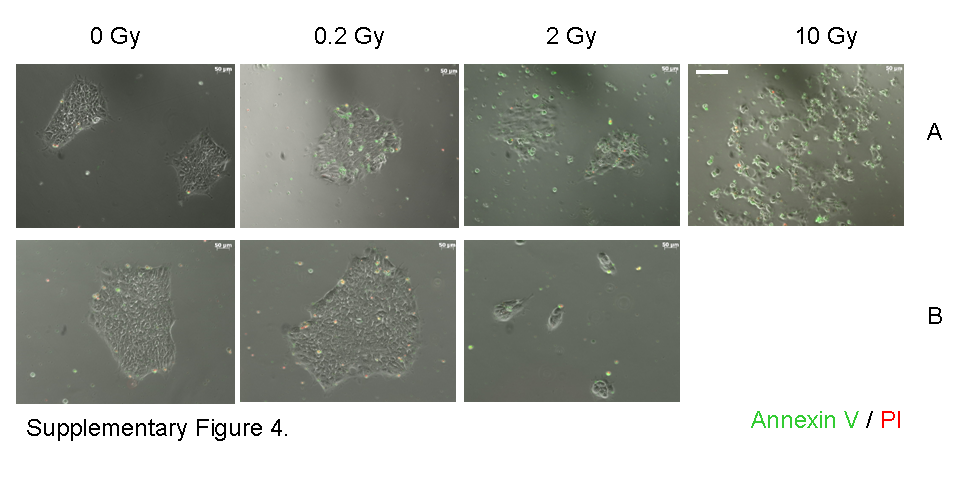

Supplement: Figure S4 — Live cell analysis of the kinetics of apoptotic cell death in directly IR-exposed hESC. Irradiated hESC were cultured either for 6 hr (A) or for 24 hr (B) post-IR. Shown in green are Annexin V-positive (apoptotic) hESC, in red - apoptotic hESC nuclei stained with PI. Scale bar in white equals 100 µm, 10× objective. (0.55 MB TIF) [file pone.0014195.s004.tif]

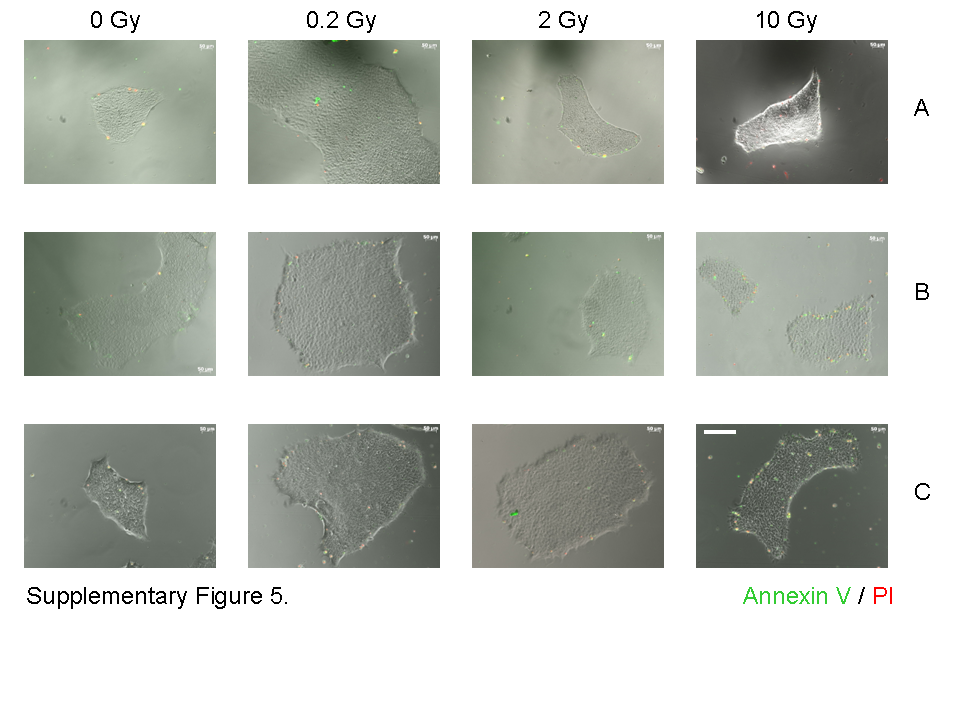

Supplement: Figure S5 — Analysis of the kinetics of apoptotic cell death in bystander hESC with media transfer. (A, B) Bystander medium harvested from directly irradiated hESC 1 hr post-exposure; (C) bystander medium harvested from directly irradiated hESC 24 hr post-exposure. Bystander hESC were cultured in conditioned medium either for 6 hr (A, C) or for 24 hr (B). Shown in green are Annexin V-positive (apoptotic) hESC, in red - apoptotic hESC nuclei stained with PI. Scale bar in white equals 100 µm, 10× objective. (0.82 MB TIF) [file pone.0014195.s005.tif]

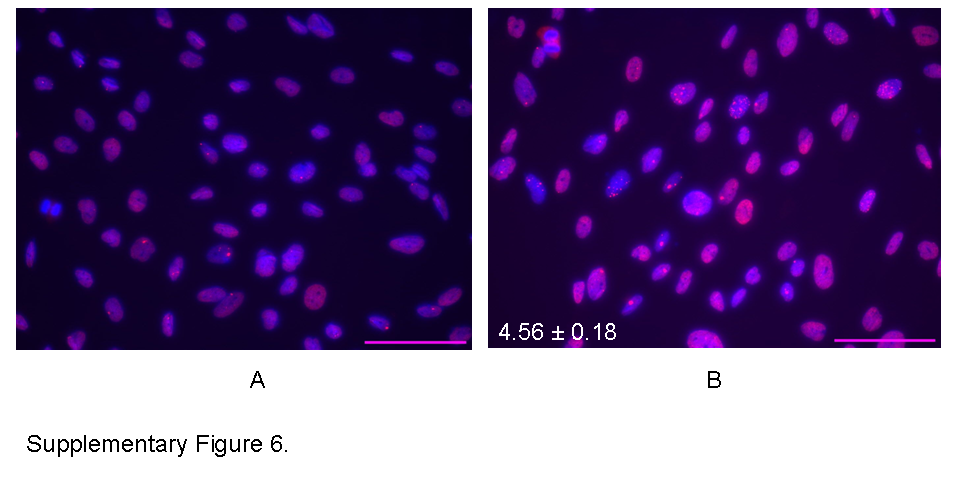

Supplement: Figure S6 — IRIF analysis of the DDR activation in bystander IMR-90 cells receiving medium conditioned for 1 hr on IMR-90 irradiated with either (A) 0 Gy, or (B) 2 Gy. Shown is the magnitude of RIBE (fraction of cells with no less than 4 IRIF per nucleus) relative to sham-irradiation, expressed as mean value ± SEM. DAPI-stained cell nuclei are in blue, and 53BP1 staining is in red. Scale bar in pink equals 50 µm, 40× objective. (0.60 MB TIF) [file pone.0014195.s006.tif]
